# Supplementary material for: Longitudinal effects of aerobic training programme on body composition in non-elite adolescent female swimmers
Source: PeerJ. 2025 May 19;13:e19456. doi: 10.7717/peerj.19456 (PMC12097237; doi:10.7717/peerj.19456)
Supplement: Supplemental Information 2 [file peerj-13-19456-s002.docx]

STROBE Statement—checklist of items that should be included in reports of studies.

|  | | | | Item No. | | Recommendation | | | Page  No. | | | | | Relevant text from manuscript | | | |  |  |
| --- | --- | --- | --- | --- | --- | --- | --- | --- | --- | --- | --- | --- | --- | --- | --- | --- | --- | --- | --- |
| **Title and abstract** | | | | 1 | | Longitudinal effects of aerobic training programme on body fat measurements in adolescent female swimmers. | | | 2 | | | | | 27 - 29 | | | |  |  |
|  |  |  |  |  |  | During a 3-year study, a group of girls practicing swimming was compared with a group not training.  There was no significant difference in the percentage of body fat between the experimental and control groups at the start of the study. This difference persisted until the final measurement after three years, at which point it became statistically significant.  The initial body fat content had a strongly negative impact on VO2max, especially in the experimental group; however, in subsequent measurements, this impact diminished and became statistically insignificant in both the experimental and control groups. | | | 5  8  8 | | | | | 161  268 - 275  275 - 283 | | | |  |  |
| Introduction | | | | | | | | | | | | | |  | | | |  |  |
| Background/rationale | | | | 2 | | Based on the literature, it can be concluded that authors are not unanimous about the effects of competitive swimming on physiological characteristics in young girls. The present study aimed to evaluate the aerobic capacity and body fat of adolescent girls as influenced by swimming training. | | | 4-5 | | | | | 143 - 146 | | | |  |  |
| Objectives | | | | 3 | | 1. How did a group of adolescent swimmers, who were not pre-selected based on specific criteria, differ in terms of aerobic capacity from a control group after three years of swimming training 2. What are the differences in body fat levels among girls who regularly trained in swimming 3. To what extent does body fat value affect aerobic capacity in both groups. State specific objectives, including any prespecified hypotheses | | | 5 | | | | | 150 - 153 | | | |  |  |
| Methods | | | | | | | | | | | | | |  | | | |  |  |
| Study design | | | | 4 | | swimming, girls, longitudinal study, body fat | | |  | | | | |  | | | |  |  |
| Setting | | | | 5 | | The research was conducted over a period of three consecutive years - between autumn 2011 and spring 2014, with measurements taken every six months between 8am and 12pm at the school attended by the respondents. | | | 5 | | | | | 178 - 179 | | | |  |  |
| Participants | | | | 6 | | (*a*) The study involved two groups of 10-year-old girls at the time of the research commencement: swimmers and control group. The experimental group consisted of 14 girls practicing swimming  Taking into account the fact of natural biological development occurring in children of this age, it is reasonable to track the changes resulting from swimming in long-term studies to which people will be recruited without preliminary selection. | | | 5  5 | | | | | 161  147 - 149 | | | |  |  |
|  |  |  |  |  |  | (*b*) The control group consisted of 14 girls who did not practice swimming | | | 5 | | | | | 166 - 168 | | | |  |  |
| Variables | | | | 7 | | 1. biological age 2. skinfold thickness over the biceps, over the triceps, beneath the inferior angle of the scapula (referred to as shoulder blade), above the iliac crest (referred to as stomach) 3. percentage of body fat (% Body fat) 4. indicator of aerobic fitness (VO_2_max) | | | 5-6  6  6  6 | | | | | 181 - 187  189 - 191  193 - 201  212 - 219 | | | |  |  |
| Data sources/ measurement | | | | 8* | | 1. biological age - calculated using formula [38]:   B.A = (body mass age + body height age + chronological age) /3  Body mass age and body height age were estimated using Pirquet's tables for girls from the Lubusz region in the Polish population (Malinowski 2005). Chronological age was calculated as the time between the participant's date of birth and the date of measurement.   1. skinfold thickness over the biceps, over the triceps, beneath the inferior angle of the scapula (referred to as shoulder blade), above the iliac crest (referred to as stomach)   All measurements were taken on the right side of the body in a standing position (Frankfurt plane) using a Harpenden skinfold caliper (M2 TOP, Käfer, Germany) with a precision of 0.1 mm.   1. percentage of body fat (% Body fat) – based on skinfolds, calculated using formula provided by Slaughter et al. (1998).   If the sum of the triceps and subscapular skinfolds was ≤35 mm:  % BF=1,33×(R+L)]-[0,013×(R+L)^2]-2,5  If the sum of the triceps and subscapular skinfolds was >35 mm:  % BF=0,546×(R+L)+9,7   1. indicator of aerobic fitness (VO_2_max) – based on The Maximal Multistage 20-m Shuttle Run Test results. Calculated using the formula provided by Léger et al. (1988):   V̇O2 max = 31,025 + 3,238 × P − 3,248 × W + 0,1536 × P × W  This test involved running back and forth over a 20-metre distance, with the pace controlled by audio signals. The participants had to complete the distance within the time allotted by the sound, which gradually shortened with each subsequent stage. The initial running speed was 8.5 km/h, and it increased by 0.5 km/h at each stage. The number of shuttle runs also increased with each stage: the first stage required seven runs at a consistent pace, the second stage required eight runs, and so on, up to the fourth stage. From stages five to eight, participants completed 10 runs, and from stages nine to thirteen, 12 runs of 20 metres were required. If a participant failed to reach the line before the next sound, their attempt was terminated. The total number of successful shuttle runs was recorded. Based on the speed at which the last stage was completed and the participant’s calendar age, maximal oxygen uptake. | | | 5-6  6  6  6 | | | | | 183-187  191 – 193  193 – 201  203 - 219 | | | |  |  |
| Bias | | 9 | | | Each measurement was performed on the same day for all people. Measurement by one and the same person. | |  | | | |  | | | | |  |  |  |  |
| Study size | | | 10 | | The number of people tested depended on the parents/guardians giving written consent to conduct the experiment. | | |  | | | | |  | | | |  |  |  |
| Quantitative variables | | 11 | | | *(a)* biological age - were handled in age [Age]  *(b)* skinfold thickness - were handled in millimetres [mm] using a Harpenden skinfold caliper  *(c)* Body fat – were handled in percentage [%] based on skinfolds measurement, calculated using formula provided by Slaughter et al. (1998)  *(d)* indicator of aerobic fitness (VO_2_max) - were handled in ml*min-^1^*kg-^1^, calculated using the formula provided by Léger et al. (1988): | | | | | 5  6  6  6 | |  | | | 183  193  193-198  213 | | | | |
| Statistical methods | | 12 | | | (*a*) To assess the significance of differences in biological age, VO2max and measures of body adiposity we performed a two-way analysis of variance (ANOVA), comparing measurements at first and sixth timepoint within the experimental and control groups, as well as between the groups. In order to determine whether measurement timepoint and swimming training interact in influencing changes in aerobic capacity and adiposity features we also examine their interaction. All analyses were conducted using stats for R (R Core Team, 2021).  To analyze the longitudinal influence of body fat percentage on changes in aerobic capacity, we employed linear mixed-effects models (LMM) to account for repeated measurements within the sample. Separate models were fitted for each group (experimental and control), allowing for comparison of the effects within and between groups.  The models were structured as follows:  VO2max=β0 + β1(Body Fatit) + β2(Timet) + β3(Body Fatit × Timet) + ui + ϵit  Where,  β0 represents the intercept (baseline aerobic capacity); β1, β2 and β3 represent the fixed effects for body fat percentage, time, and their interaction, respectively; ui represents the random intercept for each participant to account for individual variability; ϵit is the residual error.  The models were fitted using the lme4 [44] package in R (R Core Team, 2021). Marginal R2 (proportion of variance explained by fixed effects) and conditional R2 (proportion of variance explained by both fixed and random effects) were computed using the MuMIn [45] package to assess model fit. The beta coefficients (fixed effects estimates) were extracted to quantify the effects of body fat percentage and time on VO2max. Standard errors were reported for each estimate. Within the experimental group model, the VO2max of the swimmers was modelled as a function of body fat percentage, time, and the interaction between body fat and time, with random intercepts for each individual swimmer. The VO2max of the non-swimmers was modelled using the same structure to allow for comparison with the experimental group. | | | | | 7 | |  | | | 227-254 | | | | |
|  |  |  |  |  | (*b*) To assess the significance of differences in biological age, VO2max and measures of body adiposity we performed a two-way analysis of variance (ANOVA), comparing measurements at first and sixth timepoint within the experimental and control groups, as well as between the groups. In order to determine whether measurement timepoint and swimming training interact in influencing changes in aerobic capacity and adiposity features we also examine their interaction. All analyses were conducted using stats for R (R Core Team, 2021). | | | | | 7 | |  | | | 228-233 | | | | |
|  |  |  |  |  | (*c*) Explain how missing data were addressed | | | | |  | | NA | | |  | | | | |
|  |  |  |  |  | (*d*) To analyze the longitudinal influence of body fat percentage on changes in aerobic capacity, we employed linear mixed-effects models (LMM) to account for repeated measurements within the sample. Separate models were fitted for each group (experimental and control), allowing for comparison of the effects within and between groups.  The models were structured as follows:  VO2max=β0 + β1(Body Fatit) + β2(Timet) + β3(Body Fatit × Timet) + ui + ϵit  Where,  β0 represents the intercept (baseline aerobic capacity); β1, β2 and β3 represent the fixed effects for body fat percentage, time, and their interaction, respectively; ui represents the random intercept for each participant to account for individual variability; ϵit is the residual error.  The models were fitted using the lme4 [44] package in R (R Core Team, 2021). Marginal R2 (proportion of variance explained by fixed effects) and conditional R2 (proportion of variance explained by both fixed and random effects) were computed using the MuMIn [45] package to assess model fit. The beta coefficients (fixed effects estimates) were extracted to quantify the effects of body fat percentage and time on VO2max. Standard errors were reported for each estimate. Within the experimental group model, the VO2max of the swimmers was modelled as a function of body fat percentage, time, and the interaction between body fat and time, with random intercepts for each individual swimmer. The VO2max of the non-swimmers was modelled using the same structure to allow for comparison with the experimental group. | | | | | 7 | |  | | | 235-254 | | | | |
|  |  |  |  |  | (*e*) To analyze the longitudinal influence of body fat percentage on changes in aerobic capacity, we employed linear mixed-effects models (LMM) to account for repeated measurements within the sample. Separate models were fitted for each group (experimental and control), allowing for comparison of the effects within and between groups.  The models were structured as follows:  VO2max=β0 + β1(Body Fatit) + β2(Timet) + β3(Body Fatit × Timet) + ui + ϵit  Where,  β0 represents the intercept (baseline aerobic capacity); β1, β2 and β3 represent the fixed effects for body fat percentage, time, and their interaction, respectively; ui represents the random intercept for each participant to account for individual variability; ϵit is the residual error.  The models were fitted using the lme4 [44] package in R (R Core Team, 2021). Marginal R2 (proportion of variance explained by fixed effects) and conditional R2 (proportion of variance explained by both fixed and random effects) were computed using the MuMIn [45] package to assess model fit. The beta coefficients (fixed effects estimates) were extracted to quantify the effects of body fat percentage and time on VO2max. Standard errors were reported for each estimate. Within the experimental group model, the VO2max of the swimmers was modelled as a function of body fat percentage, time, and the interaction between body fat and time, with random intercepts for each individual swimmer. The VO2max of the non-swimmers was modelled using the same structure to allow for comparison with the experimental group. | | | | | 7 | |  | | | 235-254 | | | | |
|  | Results | | | | | | | | | | | | | | | | | |  |
| Participants | | 13* | | | Experimental group – 14 person included in the study completing fallow-up at each stage of study  Control group - 14 person included in the study completing fallow-up at each stage of study | | | | | 5 | |  | | | 161-162 | | | | |
| Descriptive data | | 14* | | | The experimental group included 14 swimmers who trained at student sports clubs in Czestochowa, Poland. Recruitment for the sports clubs occurred without any initial pre-selection. Upon starting the study, the girls began swimming training, though they had prior swimming skills from attending swimming lessons twice a week.  The control group consisted of 14 girls who only participated in mandatory physical education classes. Apart from their obligatory physical education lessons, the control group did not engage in any additional physical activity during the research period. | | | | | 5 | |  | | | 161 - 170 | | | | |
| Outcome data | | 15* | | | Each measurement including the studied variables, performed every six months, was collected in a separate file and marked with numbers 1-6. | | | | |  | |  | | |  | | | | |
| Main results | | 16 | | | 1. The linear regression analyses performed for each individual measurement timepoint revealed distinct patterns for swimmers and non-swimmers. At the initial measurement, swimmers exhibited a strong negative relationship between body fat percentage and VO2max, explaining 21.8% of the variance (Beta= -0.35, SE= 0.19). In the control group, the effect was much weaker, predicting 2% of the variance (Beta= -0.04, SE = 0.08). By the second follow-up, the negative impact of body fat percentage on VO2max in swimmers attenuated, with a beta of -0.24 (SE= 0.28) and R2 reduced to 6%. Interestingly, in the control group, the relationship strengthened, with a beta of -0.17 (SE= 0.09) and R2 of 24%, indicating a greater impact of body fat on VO2max. In subsequent follow-ups, the predictive power of body fat continued to weaken for both groups. By the sixth follow-up, the relationship between percentage of body fat and aerobic capacity was nonsignificant in both groups. Overall, while the percentage of body fat initially had a strong negative effect on VO2max, especially for swimmers, its influence diminished over time. | | | | | 8-9 | |  | | | 294-306 | | | | |
|  |  |  |  |  | (*b*) Report category boundaries when continuous variables were categorized | | | | |  | | NA | | |  | | | | |
|  |  |  |  |  | (*c*) If relevant, consider translating estimates of relative risk into absolute risk for a meaningful time period | | | | |  | | NA | | |  | | | | |

Continued on next page

| Other analyses | 17 | Upon visual examination of histogram plots and the skew statistics, all variables were normally distributed (Figure 1 and Table 1). Trajectories of biological age, body fat, aerobic capacity and skinfold thickness measurements throughout the duration of the study are illustrated in Figure 2 and Table 1 and compared between the experimental and control groups.  The results indicated that both at the onset of the study and after three years of training, the swimmers did not differ in biological age from their non-swimming peers (Figure 2 and Table 1). The biological age of the girls increased steadily across both groups throughout the measurement period (Figure 2). The group of girls engaged in competitive swimming for three years significantly differed in skinfold thickness from the non-training group. The analysis of variance revealed that the experimental group exhibited significantly less body fat at all four measurement sites during the initial measurement (Table 1). After three years of training, significant differences between the two groups persisted and increased with the mean differences between the initial and final measurements of 2.73mm at the shoulder blade, 5.56mm at the stomach area, 3.58mm above the biceps, and 2.52mm above the triceps (Table 1). Despite the absence of preliminary selection, the swimmers differed substantially, although not significantly, from the control group in terms of body fat percentage at the initial stage of the study (18.62% vs 24.85%), and this difference persisted through to the final measurement after three years, when it became statistically significant (17.31% vs 27.14%) (Table 1). The VO2max index significantly differed between the groups, with swimmers demonstrating greater aerobic capacity throughout the study period compared to non-swimmers (Figure 2 and Table 1). Although there was already a significant difference in aerobic capacity between the groups at the beginning of the measurements (47.48mL/kg/min vs 40.15mL/kg/min), over the three-year training period, we observed the interaction between the effects of swimming training and measurement timepoint, with the swimming group experienced a significant increase in VO2max, while the non-training group showed a significant decline in this parameter (51.30mL/kg/min vs 37.53mL/kg/min) (Figure 2 and Table 1).  Regression analyses predicting VO2max from body fat percentage across six follow-up points demonstrated distinct patterns for the experimental (swimming) and control (non-swimming) groups (Figure 3). The LMM analysis revealed that, across the 6 follow-ups, percentage of body fat explained more variation in aerobic capacity in the experimental group, compared to the control group. The marginal R2 values were estimated as 15% in the group of swimmers and 12% in controls, whereas the conditional R2 values were estimated as 66% and 82%, respectively. This discrepancy suggests that the inclusion of random effects substantially altered the predictive power of the fixed effects in the model.  The linear regression analyses performed for each individual measurement timepoint revealed distinct patterns for swimmers and non-swimmers. At the initial measurement, swimmers exhibited a strong negative relationship between body fat percentage and VO2max, explaining 21.8% of the variance (Beta= -0.35, SE= 0.19). In the control group, the effect was much weaker, predicting 2% of the variance (Beta= -0.04, SE = 0.08). By the second follow-up, the negative impact of body fat percentage on VO2max in swimmers attenuated, with a beta of -0.24 (SE= 0.28) and R2 reduced to 6%. Interestingly, in the control group, the relationship strengthened, with a beta of -0.17 (SE= 0.09) and R2 of 24%, indicating a greater impact of body fat on VO2max. In subsequent follow-ups, the predictive power of body fat continued to weaken for both groups. By the sixth follow-up, the relationship between percentage of body fat and aerobic capacity was nonsignificant in both groups. Overall, while the percentage of body fat initially had a strong negative effect on VO2max, especially for swimmers, its influence diminished over time. | 8-9 | 258-306 |
| --- | --- | --- | --- | --- |
| Discussion | | | | |
| Key results | 18 | 1. The VO2max index significantly differed between the groups, with swimmers demonstrating greater aerobic capacity throughout the study period compared to non-swimmers. over the three-year training period, we observed the interaction between the effects of swimming training and measurement timepoint, with the swimming group experienced a significant increase in VO2max, while the non-training group showed a significant decline in this parameter. 2. The group of girls engaged in competitive swimming for three years significantly differed in skinfold thickness from the non-training group. Experimental group exhibited significantly less body fat at all four measurement sites during the initial measurement. After three years of training, significant differences between the two groups increased. Despite the absence of preliminary selection, the swimmers differed substantially, although not significantly, from the control group in terms of body fat percentage at the initial stage of the study, and this difference persisted through to the final measurement after three years, when it became statistically significant. 3. While the percentage of body fat initially had a strong negative effect on VO2max, especially for swimmers, its influence diminished over time. | 8  8  8 | 275 - 283  265 – 275  294 - 306 |
| Limitations | 19 | A source of potential imprecision in the results may be the fact that in these studies examining the percentage of body fat, the diet of respondents participating in the study was not taken into account. | 10 | 347 - 350 |
| Interpretation | 20 | The results suggested that the three-year training programme contributed to an improvement in aerobic capacity.  Systematic aerobic exercise in water may inhibit the increase in body fat, consistent with other studies  Endurance training in the 10-12 age swimmer group may have gradually shaped their physique in line with the demands of this discipline, reducing body fat levels and its impact on aerobic capacity. | 9  9  11 | 332 – 334  341 – 342  394 - 396 |
| Generalisability | 21 | Swimming during the prepubescent period may be an effective means of preventing obesity | 11 | 404 - 405 |
| Other information | |  | | |
| Funding | 22 | Funded by the University |  |  |

*Give information separately for cases and controls in case-control studies and, if applicable, for exposed and unexposed groups in cohort and cross-sectional studies.
